# Assessing the Binding of Venoms from Aquatic Elapids to the Nicotinic Acetylcholine Receptor Orthosteric Site of Different Prey Models

**DOI:** 10.3390/ijms21197377

**Published:** 2020-10-06

**Authors:** Richard J. Harris, Nicholas J. Youngman, Christina N. Zdenek, Tam M. Huynh, Amanda Nouwens, Wayne C. Hodgson, David Harrich, Nathan Dunstan, José A. Portes-Junior, Bryan G. Fry

**Affiliations:** 1 Toxin Evolution Lab, University of Queensland, Biological Sciences, St. Lucia, QLD 4072, Australia; rharris2727@gmail.com (R.J.H.); christinazdenek@gmail.com (C.N.Z.); n.youngman@uq.edu.au (N.J.Y.); 2 Department of Pharmacology, Biomedicine Discovery Institute, Monash University, Clayton, VIC 3800, Australia; tlhuy3@student.monash.edu (T.M.H.); wayne.hodgson@monash.edu (W.C.H.); 3 School of Chemistry and Molecular Biosciences, University of Queensland, St Lucia, QLD 4072, Australia; a.nouwens@uq.edu.au; 4 QIMR Berghofer, Royal Brisbane Hospital, Herston, QLD 4029, Australia; david.harrich@qimrberghofer.edu.au; 5 Venom Supplies, Tanunda, SA 5352, Australia; nathan@venomsupplies.com; 6 Laboratório de Coleções Zoológicas, Instituto Butantan, São Paulo 05503-900, Brazil; portes.junior@butantan.gov.br

**Keywords:** Elapidae, venom, neurotoxicity, nicotinic acetylcholine receptors, orthosteric, allosteric

## Abstract

The evolution of an aquatic lifestyle from land dwelling venomous elapids is a radical ecological modification, bringing about many evolutionary changes from morphology to diet. Diet is an important ecological facet which can play a key role in regulating functional traits such as venom composition and prey-specific targeting of venom. In addition to predating upon novel prey (e.g., fish, fish eggs and invertebrates), the venoms of aquatic elapids also face the challenge of increased prey-escape potential in the aquatic environment. Thus, despite the independent radiation into an aquatic niche on four separate occasions, the venoms of aquatic elapids are evolving under convergent selection pressures. Utilising a biolayer interferometry binding assay, this study set out to elucidate whether crude venoms from representative aquatic elapids were target-specific to the orthosteric site of postsynaptic nicotinic acetylcholine receptor mimotopes of fish compared to other terrestrial prey types. Representatives of the four aquatic lineages were: aquatic coral snakes representative was *Micrurus surinamensis*;, sea kraits representative was *Laticauda colubrina;* sea snakes representatives were two *Aipysurus* spp. and eight *Hydrophis* spp; and water cobras representative was *Naja annulata*. No prey-specific differences in crude venom binding were observed from any species tested, except for *Aipysurus laevis,* which showed slight evidence of prey-potency differences. For *Hydrophis caerulescens*, *H. peronii*, *H. schistosus* and *M. surinamensis*, there was a lack of binding to the orthosteric site of any target lineage. Subsequent testing on the in vitro chick-biventer cervicis muscle preparation suggested that, while the venoms of these species bound postsynaptically, they bound to allosteric sites rather than orthosteric. Allosteric binding is potentially a weaker but faster-acting form of neurotoxicity and we hypothesise that the switch to allosteric binding is likely due to selection pressures related to prey-escape potential. This research has potentially opened up the possibility of a new functional class of toxins which have never been assessed previously while shedding light on the selection pressures shaping venom evolution.

## 1. Introduction

The evolution of an aquatic lifestyle (both marine and freshwater) from land-dwelling snakes requires radical morphological and ecological changes [1,2,3]. This shift to an aquatic environment has evolved on at least four independent occasions across the Elapidae family of snakes: once in the cobras (water cobra clade; *Naja annulata* and *N. christyi*), once in the coral snakes (the Amazonian coral snake; *Micrurus surinamensis*), once in the Asian elapids (sea kraits; *Laticauda* spp.), and once in the Australian elapids (sea snakes; last common ancestor of *Aipysurus* and *Hydrophis*) [4]. This change in environment has further led to major alterations in their diet preference [1], from terrestrial vertebrates such as amphibians, reptiles, mammals and birds to fish, fish eggs and marine invertebrates [5,6,7]. These changes in diet are likely to require a different venom composition and possibly pathophysiological modes of targeting in comparison to their terrestrial counterparts. For example, a diet shift can alter venom function; some species (e.g., *Aipysurus eydouxii* and *Emydocephalus* spp.) have subsequently shifted to feeding exclusively on fish eggs, consequently bringing about the atrophy and loss of their venom glands [5,8]. A focus on aquatic prey types is also likely to be the primary reason why many sea snakes have extraordinarily potent neurotoxic venoms [9], as they need to quickly immobilise fish prey that have a high escape potential. This is particularly important given that sea snakes likely lack post-envenomation scent cues in the aquatic medium, which terrestrial snakes depend upon, since their olfactory organs are highly degenerated compared to terrestrial snakes [10]. This would, in turn, likely mean that they cannot follow envenomed prey as effectively as their terrestrial relatives. Another reason for this prominent neurotoxicity in sea snake venom might be that other pathophysiological functions such as coagulotoxicity are likely not as effective on simpler/divergent circulatory systems of fish and marine invertebrates in comparison to terrestrial vertebrates as mammals and birds [11,12].

Neurotoxicity produced by elapid venoms is predominately caused by three-finger toxins (3FTxs) that target the muscle-type α-1 nicotinic acetylcholine receptor (nAChR) at the postsynaptic terminal of the neuromuscular junction [13,14,15,16]. Studies have consistently revealed that 3FTxs have a particular affinity for blocking of the α-1 orthosteric site (acetylcholine binding region) at amino acid positions 187–200, with specific key residues in this region being their primary target [17,18,19,20,21]. This mode of action causes flaccid paralysis by preventing the neurotransmitter acetylcholine (ACh) from binding, which would otherwise trigger muscular contractions [13,22]. The toxins that target the postsynaptic site are classed as α-neurotoxins [13].

3FTxs are a non-enzymatic toxin class that are ubiquitous throughout the elapids [13,14] and have been found in some colubrids [23,24,25,26] and other advanced snakes [27]. It was revealed that 3FTxs are under positive diversifying selection [28], with small amino acid changes likely allowing for the alteration in target specificity and function. The neurotoxicity of 3FTxs by targeting nAChRs seems to be the dominant pathophysiology, however, some 3FTxs have been shown to utilise different methods of neurotoxic targeting such as blocking of adrenergic receptors [29,30], acid-sensing (ASIC1) channels [31], L-type Ca^2+^ channels [32], muscarinic receptors [33,34,35], and sodium ion channels [36], as well as inhibiting acetylcholinesterase [37]. Other functions, such as coagulotoxicity [38,39,40] and cytotoxicity [41,42], are also exploited by 3FTxs. In combination with the diversity of pathophysiological targets of 3FTxs, they can also exhibit prey-specificity [23,24,25,26,43,44,45,46]. Clearly, 3FTxs are a widely diverse toxin class and, as such, venoms rich in 3FTxs are ideal to further explore their evolution, biochemistry and pharmacology.

The venom compositions of many aquatic snakes are composed mostly of neurotoxic 3FTxs—in some cases >75% of the venom composition—along with phospholipase A_2_ (PLA_2_) toxins [47,48,49,50,51,52,53]. Given the rapid onset of neurotoxic pathologies produced by many neurotoxins within these toxin classes, venoms possessing large proportions of these toxin types again suggest the need for fast immobilisation as a key selection pressure in aquatic environments.

This study initially set out to determine whether the change in ecological niche of aquatic elapids (two *Aipysurus*, eight *Hydrophis*, one *Laticauda*, one *Micrurus* and one *Naja* species) has led to the development of prey-specific venom effects, particularly to that of their likely natural prey (fish). This was conducted utilising a well-established bio-layer interferometry (BLI) assay designed to test the binding of α-neurotoxic venoms to the orthosteric site of taxa-specific nAChR mimotopes (small molecules that mimic part of the epitope) [54,55,56]. However, through unexpected initial findings, the study further addressed some new questions regarding the biochemistry and pharmacology of the venoms tested, such as the potential for allosteric targeting toxins within aquatic elapid venoms, which has never been assessed

## 2. Results and Discussion

**Figure 1:**
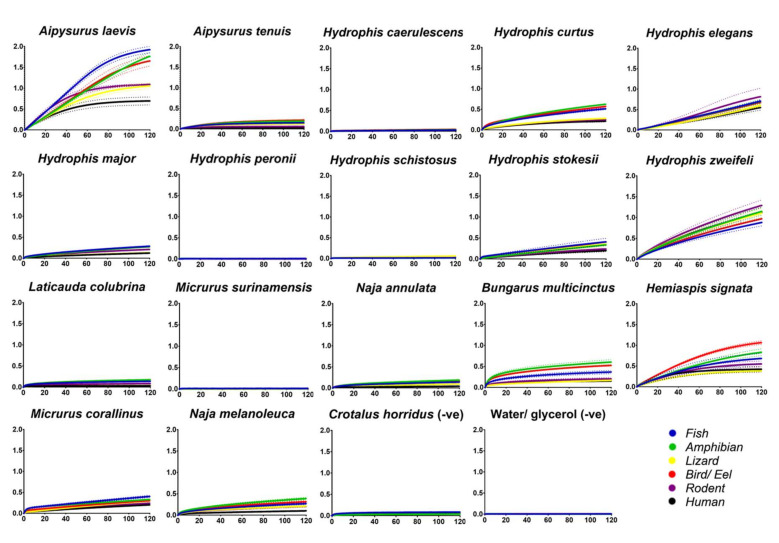
A binding comparison of crude venom from 17 species of aquatic elapids across α-1 nAChR mimotopes (fish, amphibian, lizard, bird/eel (both share the same sequence), rodent and human representatives). The two negative (-ve) controls are Crotalus horridus and 1:1 water/glycerol. Crotalus horridus was chosen as there is no evidence that this species utilises nAChR targeting neurotoxins, but will give a comparison of a venom that is rich in large non-binding toxin types (e.g., PLA_2_s, SVSPs and SVMPs [65,66]) which may cause a greater light shift than the water/glycerol -ve control. The Y-axis shows wavelength shift (nm) of the association (Ka binding step); the X-axis displays seconds (for the 120-s association period). All venoms were tested in triplicate. The dots surrounding the curve lines are error bars based on SEM values with *n* = 3. All raw data can be found in Supplementary File S1.

Screening of aquatic elapid crude venoms to determine the potential for prey-specific binding of nAChR orthosteric mimotopes revealed some interesting and unexpected facets. Firstly, addressing our initial investigation, there is no true prey-specific targeting between all the species tested (Figure 1), or at least for the prey taxa mimotopes we assessed. This is indicated by the wavelength shift curves being particularly close/overlapping for the majority of the taxa mimotopes. *Aipysurus laevis* binds slightly more to the fish than the other taxa mimotopes, however true prey-selective venom would either be disproportionately higher than all other mimotope values, as seen with *Ophiophagus hannah* preferentially targeting the snake mimotope [55,56], or it would be the only mimotope that showed binding. In the case of *A. laevis*, the fish, amphibian and bird/eel mimotope curves are clustered together (Figure 1), which is not indicative of preferential prey binding. This is also consistent with the fish orthosteric site, differing from the amphibian by only two amino acids, one of which (D for E) is a biochemically conserved substitution and unlikely to change binding. Similarly, the fish differed from the bird/eel by only one amino acid (bird orthosteric site is the same sequence as the representative eel sequence). Thus, the similar level of binding was consistent with the biochemical similarities of these three orthosteric sites.

Further to this, the α-1 nAChR (chrna1) sequence for the ‘eel’ representative is a freshwater species (*Gymnotus electricus;* Anguilliformes (P09688)), which would not be a prey target for the marine aquatic elapids, and thus might not be truly representative of true prey. Although this sequence seems conserved between other freshwater eel lineages (e.g., *Mastacembelus armatus;* Synbranchiformes (A0A3Q3LHR6)), there are no sequenced marine representatives with which to compare. Thus, it is uncertain if the freshwater *Gymnotus electricus* sequence remains conserved across other marine families, including its closely related marine anguilliform relatives which are prey to many sea snakes [6]. Evidence has suggested there is resistance within the marine eels *Gymnothorax moringa, G. hepaticus* and *G. undulatus* (Gymnotiformes) to *Laticauda colubrina* and *A. laevis* venom [57,58], whereas *Anguilla rostrata* (Anguilliformes) were not resistant [57]. However, it is unclear if this resistance is seen at the nAChR orthosteric site, similarly to other species’ resistance to elapid 3FTxs [59,60,61]. Once more sequences for marine eel representatives become publicly available, future research should endeavour to test sea snake venom against these sequences to assess their binding and potential resistance.

Some aquatic elapids are also known to feed on marine invertebrates [6,7], which no mimotope representatives were tested against in this study. Invertebrates may show a different binding and/or prey-selective targeting by some of the aquatic elapid venoms, particularly the species that have relatively low binding to the BLI assay (Figure 1). Invertebrate-specific venom has been found within some species of the viper genus *Echis* [62] and, given the propensity for 3FTxs to be extremely selective [23,25,26,44], it is possible that some of these aquatic elapids could contain invertebrate-specific toxins. Future work should test marine invertebrate prey nAChR orthosteric mimotopes against these aquatic elapid venoms.

In the present study, *Aipysurus laevis* venom had a very high binding affinity in comparison to all other venoms tested and is one of the highest binding of all elapid venoms tested on this BLI assay to date [54,55]. This species is a generalist feeder, predating on a large diversity of fish (>17 different families), fish eggs and marine invertebrates (e.g., cephalopods, crustaceans) [6,7,63]. This type of diet may be a factor explaining why *A. laevis* venom would seem more broadly potent across the different taxa types, since needing to immobilise a broader taxonomical variety of prey types appears to require less specific and more general acting 3FTxs [64]. These data may further support the idea that diet breadth can mediate venom potency in relation to prey-specificity [64].

Some species, such as *A. tenuis*, *H. stokesii*, *H. major*, *L. colubrina* and *N. annulata*, although having toxins that bound to the mimotopes, were relatively low-binding in comparison to other species as *A. laevis, H. elegans, H. curtus* and two of the terrestrial comparison species; *B. multicinctus* and *Hemiaspis signata*. This low binding has been previously suggested to potentially be due to the proportions of 3FTxs within the venom [55], i.e., a low concentration of 3FTx might give a weaker binding detection than a venom with high concentrations of 3FTxs. However, this does not seem likely in the case of many of these aquatic elapids, as their venoms have been shown to be dominated by 3FTxs in high proportions [47,48,49,50,51]. Furthermore, the venoms of *Hydrophis caerulescens*, *H. peronii*, *H. schistosus* and *M. surinamensis* did not bind to any mimotope (Figure 1). This is despite previous studies on some of these same species showing postsynaptic neurotoxicity [9] and with clinical reports showing predominantly neurotoxic symptoms [67,68]. Thus, the low/lack of binding may be that the predominant neurotoxic targeting of these venoms is either at the presynaptic junction or that the toxins are targeting allosterically (binding to the nAChR at a site other than the orthosteric). Consequently, while some venoms did not bind to the orthosteric site, this does not preclude other mechanisms of neurotoxicity being present.

**Figure 2:**
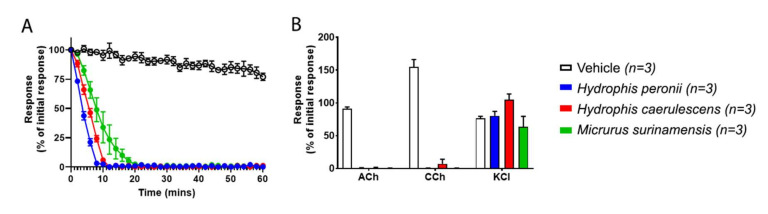
In vitro neurotoxicity using the chick-biventer cervicis nerve muscle preparation (CBCNM); (**A**) Inhibition of indirect twitches in the CBCNM by Hydrophis peronii (blue), H. caerulescens (red) and Micrurus surinamensis (green). Vehicle (white) is the control. (**B**) The effect of venoms on the contractile response to exogenous agonists acetylcholine (ACh), carbachol (CCh), and potassium chloride (KCI). All venoms were tested in triplicate (*n* = 3).

Thus, to determine if the venoms were neurotoxic in a presynaptic or allosteric manner, an in vitro neurotoxicity chick-biventer cervicis nerve muscle preparation (CBCNM) assay was used to assess the venoms that lacked orthosteric binding (Figure 1). The results from the CBCNM assay indicate that the venoms of *H. caerulescens, H. peronii* and *M. surinamensis* rapidly abolished twitch response (Figure 2A), by acting upon nAChRs at the postsynaptic junction, as indicated by the lack of response to exogenous ACh and CCh (Figure 2B). Although *H. schistosus* did not bind to the postsynaptic orthosteric site (Figure 1), there was not enough crude venom to test on CBCNM. However, *H. schistosus* has been previously shown to exhibit potent postsynaptic nAChR activity [9].

Since the BLI assay to test orthosteric postsynaptic activity did not detect any binding for *H. caerulascens*, *H. peronii* and *M. surinamensis* (Figure 1), and the CBCNM assay, which can detect differential targeting of the post- and presynaptic nerve terminal, revealed potent postsynaptic nAChR action (Figure 2), the most parsimonious explanation for these results is that the venoms are indeed acting allosterically. To date, only one allosterically targeting 3FTx has been identified, however, this only seems to be allosteric toward muscarinic receptors (mAChRs) [35]. Given this, and the notion that there is a wide diversity of 3FTx functional activities, it is no surprise that allosteric nAChR inhibition might have also evolved. Furthermore, it might be that low binding venoms (Figure 1) may indeed also contain these allosteric targeting toxins in combination with orthosteric. For example, *N. annulata,* which bound only weakly to the orthosteric site (Figure 1) has been shown previously to be potently neurotoxic at the postsynaptic junction when tested on the CBCNM [69]. Therefore, while this species does not bind strongly to the orthosteric site, it may be likely that other toxins are present in the venom that bind to the allosteric site of the postsynaptic nAChR, causing an overall greater potency.

Future research should assess if these venoms are indeed allosteric by utilising a two-electrode voltage-clamp assay. Further, a similar method, as previously mentioned [70], in combination with the BLI assay used here to test a series of overlapping mimotopes which correspond to the whole length of the α-1 nAChR, could also test for allosteric binding.

**Figure 3:**
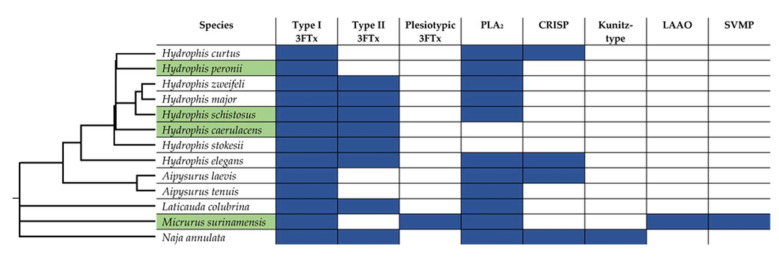
LC-MS/MS results highlighted by their toxin class representative matches from ProteinPilot™ v. 5.0. Toxin family abbreviations are as follows: Type I 3FTx, short-chain three-finger toxin; Type II 3FTx, long-chain three finger toxin; Plesiotypic 3FTx, plesiotypic three-finger toxin; PLA2, phospholipase A_2_; CRISP, cysteine-rich secretory peptide; Kunitz-type; kunitz-type protease inhibitor; LAAO, L-amino acid oxidase; SVMP, snake venom metalloprotease. Blue rectangles indicate the toxin type was matched from the LC-MS/MS results. Species names highlighted in green were species that did not bind orthosterically to postsynaptic nAChR mimotopes (Figure 1). Phylogeny was taken from Timetree.org and adjusted using recent phylogenetic data [4]. All LC-MS/MS raw data output from ProteinPilot™ v. 5.0 can be found in Supplementary File S2.

Venom compositions were determined using LC-MS/MS to observe any differences or similarities between the venoms of the species that bound allosterically and the venoms that bound to the orthosteric site. The only commonality between the LC-MS/MS results from the species that show possible allosteric targeting is that they all contain Type I 3Ftxs (Figure 3). Comparisons between Type I and Type II 3Ftxs highlight that they display differential targeting toward nAChRs [13]. For example, Type I 3FTxs are much faster acting than Type II, associating 6–7 fold faster and dissociating 5–9 fold faster, indicating a faster but weaker binding of Type I [13]. Type I 3FTxs are also more reversible than Type II [13]. It might be that these differences in binding between Type I and Type II 3FTxs are suggestive of allosteric targeting by Type I 3FTxs. It is possible that there is a biochemical trade-off with binding allosterically, with it being a quicker acting toxin but weaker in binding strength. The evolution of these faster acting allosteric toxins is likely to occur given a strong selection pressure to immobilise prey with either a high escape potential or a greater risk of prey handling.

A comprehensive study on the diets of marine snakes [6] revealed that *H. caerulescens* and *H. peronii* feed predominantly on Trypauchenidae species, which are burrowing gobies. This predominant diet of burrowing fish might be enough of a selective pressure to push the neurotoxic venom of these sea snakes to allosteric binding, compared to that of more generalist feeders. Burrowing fish would certainly have a higher escape potential than other prey. Similarly, *H. schistosus* is thought to feed predominantly on ariid and plotosid catfish [6,7], which are known for their defensive spines associated with venom [71,72]. Again, it might be that faster acting (possibly allosteric) neurotoxins are more beneficial for use against these prey types, since there is a greater risk of injury due to defensive envenomation. Therefore prey retaliation is another potential shaping pressure for aquatic venoms, as has been suggested for terrestrial lineages such as *Calliophis bivirgatus* feeding upon other venomous snakes [36].

In light of this, it is likely that many of the species with low orthosteric binding might also contain allosteric toxins, some having previously been shown to potently target the postsynaptic junction on CBCNM (e.g., *N. annulata* [69] and *L. colubrina* [9]).

Clearly, there is a vast gap in the literature regarding the questions posed here, particularly the presence of potential allosteric nAChR toxins and how, if at all, these differ functionally to the orthosteric nAChR toxins in their binding efficiency and immobilisation speed.

In summary, this research set out to assess the potential for prey-specific targeting of aquatic elapid venoms to that of the nAChR orthosteric site of a plethora of taxa types. During the investigation, many unexpected results posed further research questions concerning the functional targeting of the neurotoxic components of some aquatic elapid venoms. No true prey-specificity was observed, but some species, such as *Aipysurus laevis,* showed marginal prey-selectivity with fish, amphibian and bird/eel targets as its highest binding targets. However, due to the use of broad taxonomic representative mimotopes (which for some are fairly conserved across taxa groups), there is still the possibility that prey-specificity may occur. Some aquatic elapids were either low-binding or did not bind to the nAChR orthosteric mimotopes, which led the authors to hypothesise the presence of either presynaptic or allosteric targeting toxins. The venoms of species that did not bind to the BLI assay were thus further tested utilising the CBCNM assay to assess if they target presynaptically or are allosteric binders. The results indicated that their venoms blocked postsynaptic nAChRs and thus target these receptors allosterically. This further suggests that allosteric binding toxins may play a more prominent role in aquatic elapid neurotoxicity than previously realised.

Future research should endeavour to assess the venom binding of aquatic elapids to that of mimotope sequences that are species-specific to the diet of these snakes. Further work also is needed to assess if the venoms of some species do indeed contain allosteric nAChR targeting toxins and to further attempt to isolate and characterise them. This would potentially shed light on a new functional class of snake venom toxins with exciting new avenues for both evolutionary and pharmacological fields.

## 3. Materials and Methods

### 3.1. Venom Collection and Preparation

Pooled venoms were obtained from the pre-existing, long-term cryogenic research sample collection of the Toxin Evolution Lab, with no animal use specifically for this project except for the *Micrurus* species which were wild-collected under the conditions of ICMBio permits 57585 and 66597 linked to animal ethics approval from the Instituto Butantan Ethics Committee CEUA n° 4479020217 (issued on 22 March 2017) and CEUA no 3159250919 (issued 23 October 2019), respectively. All venom samples were lyophilised and reconstituted in double deionised water (ddH_2_O), and then centrifuged (4 °C, 10 min at 14,000 relative centrifugal force (RCF)). The supernatant was then made into a working stock (1 mg/mL) in 50% glycerol to prevent freezing at −20 °C. The concentrations of working stocks were determined in triplicate using a NanoDrop 2000 UV-Vis Spectrophotometer (Thermo Fisher, Sydney, Australia) at an absorbance wavelength of 280 nm.

### 3.2. Mimotope Production and Preparation

Following methods from a previously developed assay [55,56], a 13–14 amino acid mimotope of the vertebrate α-1 nAChR orthosteric site was synthesised by GenicBio Ltd. (Shanghai, China), designed upon specification obtained from chrna1 sequences from UniProt and GenBank databases.

Accession codes for the amino acid sequences of the α-1 nAChR orthosteric site for each taxa were as follows: fish α-1 (uniprot P02710), amphibian α-1 (uniprot F6RLA9), lizard α-1 (genbank XM_015426640), bird/eel α-1 (uniprot E1BT92 and uniprot P09688), rodent α-1 (uniprot P25108), human α-1 (uniprot P02708).

The Cys–Cys of the native mimotope was replaced during peptide synthesis with Ser-Ser to avoid uncontrolled postsynthetic thiol oxidation. The Cys–Cys bond in the nAChR binding region does not participate directly in analyte–ligand binding [20,73,74], thus, replacement to Ser-Ser is not expected to have any effect on the analyte–ligand complex formation. However, the presence of the Cys–Cys bridge is key in the conformation of the interaction site of whole receptors [75]. As such, we suggest that direct comparisons of kinetics data, such as Ka or KD, between nAChR mimotopes and whole receptor testing should be avoided, or at least approached with caution. Mimotopes were further synthesised to a biotin linker bound to two aminohexanoic acid (Ahx) spacers, forming a 30 Å linker.

Mimotope dried stocks were solubilised in 100% dimethyl sulfoxide (DMSO) and diluted in ddH_2_O at 1:10 dilution to obtain a stock concentration of 50 µg/mL in 10% DMSO. Stocks were stored at −80 °C until required.

### 3.3. Biolayer Interferometry (BLI)

Full details of the developed assay, including a full methodology and data analysis, can be found in the validated protocol [56] and further data using this protocol [54,55]. In summary, the BLI assay was performed on the Octet Red 96 system (ForteBio, Fremont, CA, USA). Venom (analyte) samples were diluted at 1:20 (a final experimental concentration of 50 µg/mL per well). Mimotope aliquots were diluted at 1:50 (a final concentration of 1 µg/mL per well). The assay running buffer was 1X DPBS with 0.1% BSA and 0.05% Tween-20. Prior to experimentation, Streptavidin biosensors were hydrated in the running buffer for 30–60 min, whilst on a shaker at 2.0 revolutions per minute (RPM). The dissociation of analytes occurred using a standard acidic solution glycine buffer (10 mM glycine (pH 1.5–1.7) in ddH_2_O). Raw data are provided in Supplementary File S1. All data obtained from BLI on Octet Red 96 system (ForteBio) were processed in accordance with the validation of this assay [56]. The association step data (in triplicate) were obtained in an Excel.csv file extracted from raw outputs of the Octet Red 96 system and then imported into Prism8.0 software (GraphPad Software Inc., La Jolla, CA, USA) and graphs were produced.

### 3.4. Sample Preparation for Mass Spectrometry (LC-MS/MS)

Powdered venom samples were reduced and alkylated. The protocol was as follows: 20 µL of 8M urea was added to 5 µg of powdered venom and vortexed. A total of 10 µL of 15mM dithiothreitol (DTT) was further added, vortexed and then the sample incubated at 56 °C for 30 min. The sample was then cooled to room temperature (RT) and 10 µL of 100mM iodoacetamide (IAA) added and then further incubated at RT for 30 min with reduced light. A further 10 µL of 15mM DTT was added and incubated for 30 min at RT. 50 µL of 40mM ammonium bicarbonate (ABC) was added, followed by 10 µL of trypsin solution (trypsin w/ 5% 1mM hydrochloric acid (HCL) and 95% ABC) on ice. The samples were then incubated at 37 °C for 24 h and then further lyophilised.

The lyophilised reduced and alkylated venom samples were brought up in 0.1% triflouric acid (TFA). The samples were filtered via Millipore ZipTips^®^ using the following protocol: tips were wetted with 100% acetonitrile (ACN) (3 × 10 μL) then equilibrated with 10 μL 5% ACN/0.1% TFA (3 × 10 μL). Samples were then loaded and pipetted up and down twice the volume of sample. Tips were then washed with 5% ACN/0.1% TFA (3 × 10 μL). Samples were eluted with 80% ACN/0.1% TFA (10 μL) in a new tube. Samples were then lyophilised and later resuspended in 20 μL of 5% ACN/0.1% TFA for LC-MS/MS.

### 3.5. Mass Spectrometry (LC-MS/MS)

Samples had an injection volume of 6 µL and were separated using reversed-phase chromatography on a Dionex Ultimate 3000 RSLC nano-system. Using a flow rate of 30 µL/min, samples were desalted on a Thermo PepMap 100 C18 trap (0.3 × 5 mm, 5 µm) for 5 min, followed by separation on an Acclaim PepMap RSLC C18 (150 mm × 75 µm) column at a flow rate of 300 nL/min. A gradient of 5–50% buffer B over 40 min, 50–98% B over 3 min, and held at 98% B for 6 min was used, where buffer A = 1% ACN/0.1% FA and buffer B = 80% ACN/0.1% FA was used to separate peptides. Eluted peptides were directly analysed on an Orbitrap Elite mass spectrometer (Thermo) using an NSI electrospray interface. Source parameters included a capillary temperature of 275 °C; S-Lens RF level at 63%; source voltage of 2.1 kV and maximum injection times of 200 ms for MS and 150 ms for MS2. Instrument parameters included an FTMS scan across m/z range 350–1800 at 60,000 resolution followed by CID fragmentation of the top 10 peptides in the ion trap. Dynamic ion exclusion was employed using a 15 s interval. Charge state screening was enabled with rejection of +1 charged ions and monoisotopic precursor selection enabled.

Data were converted to mascot generic format (mgf) using the msConvert software (ProteoWizard, v3.0) and searched using Protein Pilot™ v. 5.0 (Sciex). An Elapidae and Hydrophiidae protein database was downloaded from UniProt and the data were matched against toxin from these databases in ProteinPilot™ v5.0. Raw data output from ProteinPilot™ v. 5.0 is accessible in Supplementary File S2.

### 3.6. Isolated Chick-Biventer Cervicis Nerve-Muscle Preparation

Chicks aged 4 to 10 days were euthanized with CO_2_ (animal ethics MARP/2017/147 was approved by Monash University Ethics Committee on 12 May 2017). After dissection, the biventer cervicis nerve muscle (CBCNM) preparations were mounted under 1 g tension in 5 mL organ baths containing physiological salt solution (NaCl, 118.4 mM; KCl, 4.5 mM; MgSO4, 1.2 mM; KH2PO4, 1.2 mM; CaCl_2_, 2.5 mM; NaHCO_3_, 25 mM; and glucose, 11.1 mM). Organ baths were maintained at 34 °C and bubbled with carbogen (95% O_2_; 5% CO_2_). Electrodes were placed around the tendon of the biventer muscle and electrical stimulation at the motor nerve (0.2 ms duration, 0.1 Hz, supramaximal V) using a Grass S88 stimulator (Grass Instruments, Quincy, MA, USA) evoked indirect twitches. Selective stimulation of the nerve was confirmed by the abolition of twitches with d-tubocurarine (10 µM), a nAChR competitive antagonist. Tissues were then washed repeatedly with physiological salt solution to restore twitch responses to nerve stimulation. The stimulation was ceased, and the contractile responses to acetylcholine (ACh, 1mM for 30 s), carbochol (CCh, 20 µM for 60 s), and potassium chloride (KCl, 40 mM for 30 s) were obtained and recorded. The organ bath was then washed, and electrical stimulation was resumed and maintained for 30 min to allow the preparation to equilibrate. Venom (10 µg/mL) was added to the organ bath and the twitch height was recorded until the abolition of twitch response or was stopped after a 1 h period. The stimulator was turned off again and the bath was washed. Contractile responses to ACh, CCh and KCl were obtained again to compare with responses prior to venom addition. The twitch responses to electrical stimulations and contractile responses to agonists (ACh, CCh, and KCI) were measured using a Grass FT03 force displacement transducer (Grass Instruments, Quincy, MA, USA) and recorded on a PowerLab system (ADInstruments Pty Ltd., Bella Vista, NSW, Australia). The data were then converted into figures using Prism8.0 software (GraphPad Software Inc., La Jolla, CA, USA).

## Supplementary Materials

Supplementary materials can be found at https://www.mdpi.com/1422-0067/21/19/7377/s1.

## Abbreviations

nAChR	Nicotinic acetylcholine receptor
mAChR	Muscarinic acetylcholine receptor
3FTx	Three-finger toxin
PLA_2_	Phospholipase A_2_
CRISP	Cystine -rich secretory protein
Kunitz-type	Kunitz-type protease inhibitor
LAAO	L-Amino acid oxidase
SVMP	Snake venom metalloprotease
SVSP	Snake venom serine protease
BLI	Biolayer interferometry
CBCNM	Chick-biventer cervicis nerve muscle
ACh	Acetylcholine
CCh	Carbochol
KCl	Potassium chloride
